# Choosing between ticagrelor and clopidogrel following percutaneous coronary intervention: A systematic review and Meta-Analysis (2007–2017)

**DOI:** 10.1097/MD.0000000000012978

**Published:** 2018-10-26

**Authors:** Wenjun Guan, Hongtao Lu, Keping Yang

**Affiliations:** Department of Cardiology, Jingzhou Central Hospital, The Second Clinical Medical College, Yangtze University, Jingzhou, Hubei, China.

**Keywords:** clopidogrel, dual antiplatelet therapy, major bleeding, minor bleeding, mortality, percutaneous coronary intervention, stent thrombosis, ticagrelor

## Abstract

**Background:**

Limitations have been observed with the use of clopidogrel following percutaneous coronary intervention (PCI) indicating the urgent need of a more potent anti-platelet agent. We aimed to compare the efficacy and safety of ticagrelor versus clopidogrel following PCI.

**Methods:**

Online databases were searched for relevant studies (published between the years 2007 and 2017) comparing ticagrelor versus clopidogrel following coronary stenting. Primary outcomes assessed efficacy whereas secondary outcomes assessed safety. Odds ratios (OR) with 95% confidence intervals (CIs) based on a random effect model were calculated and the analysis was carried out by the RevMan 5.3 software.

**Results:**

A total number of 25,632 patients with acute coronary syndrome (ACS) [12,992 patients with ST segment elevation myocardial infarction (STEMI) and 14,215 patients with non-ST segment elevation myocardial infarction (NSTEMI)] were included in this analysis, of whom 23,714 patients were revascularized by PCI. Results of this analysis did not show any significant difference in all-cause mortality, major adverse cardiac events (MACEs), myocardial infarction, stroke and stent thrombosis observed between ticagrelor and clopidogrel with (OR: 0.83, 95% CI: 0.67–1.03; *P* = .09), (OR: 0.64, 95% CI: 0.41–1.01; *P* = .06), (OR: 0.77, 95% CI: 0.57–1.03; *P* = .08), (OR: 0.85, 95% CI: 0.57–1.26; *P* = .42) and (OR: 0.70, 95% CI: 0.47–1.05; *P* =.09).

However, ticagrelor was associated with a significantly higher minor and major bleeding with (OR: 1.57, 95% CI: 1.30–1.89; *P* = .00001) and (OR: 1.52, 95% CI: 1.01–2.29; *P* = 0.04) respectively. Dyspnea was also significantly higher in the ticagrelor group (OR: 2.64, 95% CI: 1.87–3.72; *P* = .00001).

**Conclusion:**

Ticagrelor and clopidogrel were comparable in terms of efficacy in these patients with ACS. However, the safety outcomes of ticagrelor should further be investigated.

## 1 Introduction

Dual antiplatelet therapy (DAPT) with aspirin and clopidogrel is considered as the key element to prevent stent thrombosis following percutaneous coronary intervention (PCI) with drug-eluting stents (DES).^[1]^ In order to prevent long-term recurrent events and stent thrombosis in patients with acute coronary syndrome (ACS) who are treated by DES, DAPT is usually recommended for at least 1 year,^[2]^ thereafter, only aspirin should continually be used as a measure of secondary prevention. However, since limitations such as persistent hypo-responsiveness to clopidogrel have been observed in patients with Type 2 Diabetes Mellitus (T2DM) even with a higher dosage,^[3]^ and because of the fact that higher adverse clinical outcomes have been observed with the concomitant use of clopidogrel and proton pump inhibitor (PPI),^[4]^ a more powerful anti-platelet drug was indeed required to replace clopidogrel.

Recently, ticagrelor, also known as AZD6140, the first reversibly binding oral, direct-acting P2Y12 receptor antagonist, with a faster onset and a greater inhibiting effect on platelets, has shown to be beneficial in patients with ACS.^[5]^

In this analysis, we aimed to systematically compare the efficacy and safety between ticagrelor and clopidogrel following PCI, using a large number of patients which were extracted from recent 10-year publications (2007–2017).

## 2 Methods

### 2.1 Data sources and search strategy

EMBASE, PubMed and the Cochrane library were searched for relevant publications (between the years 2007 and 2017) comparing ticagrelor with clopidogrel following coronary stenting.

The following searched terms or phrases were used: “ticagrelor and clopidogrel”. The term “percutaneous coronary intervention” was also included in this search strategy.

In addition, official websites of highly qualified journals which were expected to publish studies related to this particular topic, for example, the New England Journal of Medicine, The Lancet, PLOS Medicine, the Journal of the American College of Cardiology, Scientific Reports and Circulation were also searched for relevant studies.

### 2.2 Inclusion and exclusion criteria

Studies were included if:

(1) They compared ticagrelor with clopidogrel (single dose or double dose clopidogrel) in patients with coronary artery disease (CAD)/ACS.
(2) They reported adverse clinical outcomes (assessing efficacy or safety) or adverse drug events as their endpoints during any follow-up period after coronary stenting.

Studies were excluded if:

(1) They did not compare ticagrelor with clopidogrel, but instead, compared ticagrelor with prasugrel or clopidogrel versus prasugrel.
(2) They did not report adverse outcomes or adverse drug reactions which were associated with ticagrelor and clopidogrel as their clinical endpoints.
(3) They only reported platelet reactivity as their clinical endpoints.
(4) They reported data which could not be used in this current meta-analysis.

### 2.3 Primary outcomes which assessed efficacy included:

- All-cause mortality;
- Stroke;
- Myocardial infarction (MI);
- Stent thrombosis;
- Major adverse cardiac events (MACEs) (consisting of death, MI, revascularization and/or stroke).

### 2.4 Secondary outcomes which assessed safety included:

- **Major bleeding** including intraocular bleeding resulting in complete blindness or visual loss, or bleeding that resulted in a drop in the hemoglobin level of ≥ 3 g per deciliter but < 5 g per deciliter or bleeding that required blood transfusion of 2 to 3 units of red cells, was defined as bleeding that led to a significantly high level of disability.
- **Minor bleeding** was defined as mild bleeding that did not require intervention or bleeding that required intervention but did not satisfy the criteria for major bleeding or bleeding that was so mild to be compared with major bleeding.
- **Life-threatening bleeding** which included intracranial bleeding, intra-pericardial bleeding with cardiac tamponade, a decrease in hemoglobin level of 5.0 g or more per deciliter, or bleeding that required blood transfusion of at least 4 units of red cells, and bleeding which resulted in severe hypotension or hypovolemic shock and bleeding requiring immediate intervention (surgery) was defined as the most fatal bleeding.
- **Adverse drug events** including dyspnea, bradycardia, diarrhea, ventricular tachycardia, and drug discontinuation especially due to dyspnea.

In this analysis, the follow up time period was from day 1 to 12 months following coronary stenting/use of antiplatelet medications.

The primary (efficacy) and secondary (safety) outcomes, as well as the follow-up time periods reported in all the studies, have been listed in Tables 1 and 2.

**Table 1:**
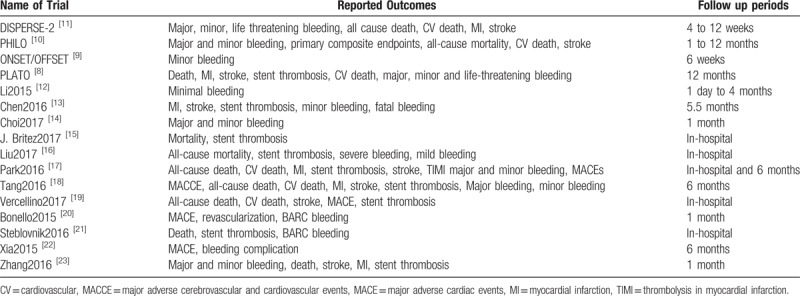
Reported adverse clinical outcomes (efficacy and safety outcomes).

**Table 2:**
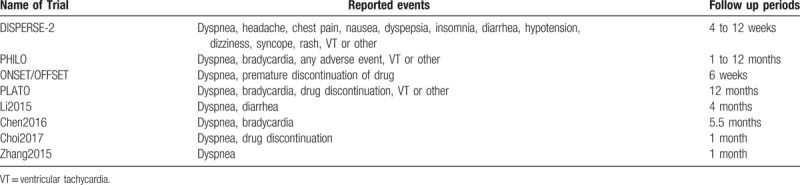
Reported adverse drug events (safety outcomes).

### 2.5 Data extraction

WG, HL, and KY independently reviewed the data and then assessed the eligibility features and methodological quality of the trials/studies which were considered relevant to this analysis. Information regarding the author surnames, year of publication, period of patients’ enrollment of the respective trial/study, region where the study was carried out, the total number of patients who were treated by ticagrelor and clopidogrel respectively, the baseline characteristics of the patients who were involved, and the reported clinical outcomes assessing efficacy, safety and adverse drug reactions were systematically extracted.

The bias risk was assessed in accordance to the Cochrane Collaboration.^[6]^ Scores ranging from 0 to 12 were allotted to represent the quality of the trials. A low risk of bias was allotted a score of 2, unclear bias risk was allotted a score of 1 whereas a high risk of bias was allotted a score of 0. Scores were given to each of the 6 components which were recommended by the Cochrane Collaboration (Table 3).

**Table 3:**
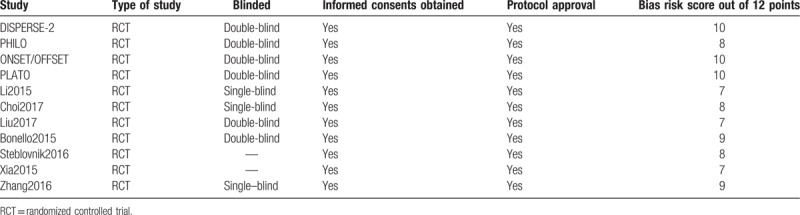
Main features of the trials and the bias risk assessment.

### 2.6 Assessment of heterogeneity reported bias and statistical analysis

The Preferred Reporting Items for Systematic Reviews and Meta-Analyses (PRISMA) guideline was considered relevant for this meta-analysis.^[7]^

Heterogeneity which was an important feature in this analysis was assessed by 2 very basic statistical techniques:

Primarily by the Cochrane Q-statistic test (*P* < .05 was considered statistically significant; statistically supporting the drug which is being favored) and secondly by the I^2^-statistic test which was obtained following the subgroup analyses. A low value of I^2^ indicated a low heterogeneity whereas an increased heterogeneity was represented by a high I^2^ value.

A random-effects model was preferred for this analysis since the included studies were performed at different times in different geographic locations and consisted of heterogeneous populations using different selection criteria.

Sensitivity analysis was carried out by a “leave-1-out” method whereby each study (for example the PLATO Trial) was excluded 1 by 1, and the differences which were obtained in results were observed and reported. Sensitivity analysis was also carried out based on the follow-up time period whereby all the studies with a shorter follow-up time period were separately analyzed from the studies with longer follow-up time periods and any significant difference in the results was noted.

Publication bias which could possibly be present was estimated by assessing funnel plots.

We calculated odds ratios (OR) and 95% confidence intervals (CIs) which were generated through the RevMan 5.3 software.

### 2.7 Ethics

Ethical committee or medical institutional board approval was not required for systematic reviews and meta-analyses.

## 3 Results

### 3.1 Searched outcomes

**Figure 1:**
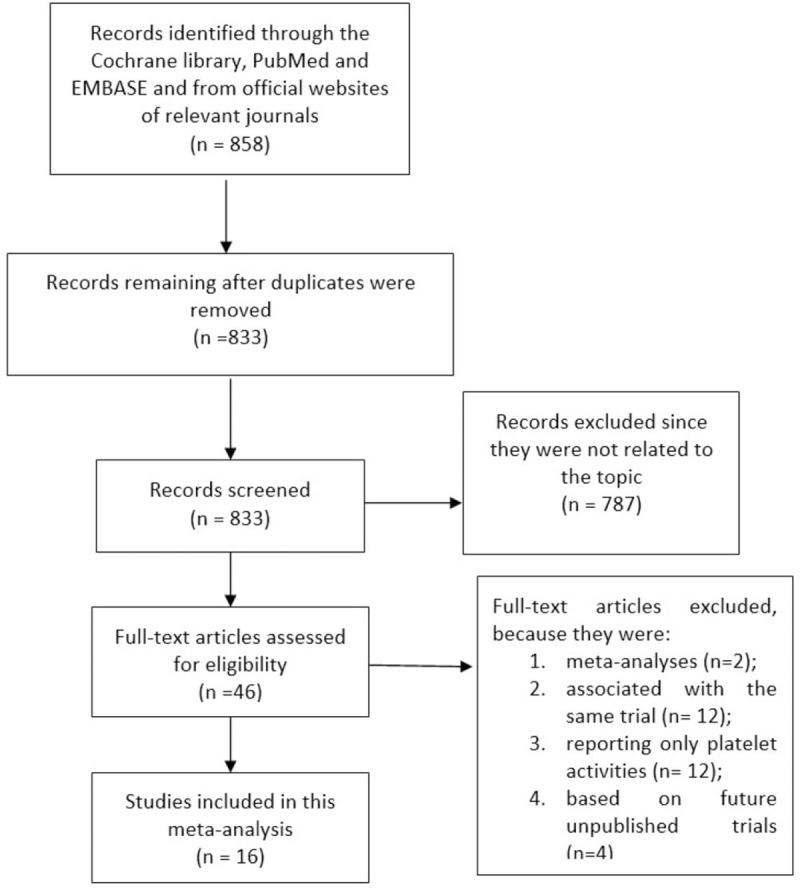
Flow diagram representing the study selection for this analysis.

Eight hundred forty-two (842) articles were obtained from the searched databases. An additional 16 publications were obtained from official websites of relevant journals. Seven hundred eighty-seven (787) articles were not related to the scope of this research and were therefore eliminated. In addition, 25 duplications (repeated studies) were also directly eliminated. Forty-six (46) full-text articles were finally reviewed for eligibility. Two (2) more article were eliminated since they were meta-analyses. A further 12 articles were eliminated since they were associated with the same PLATO trial whereas another 12 articles were eliminated since they only reported platelet activity as their clinical endpoints. At last, a further 4 articles were eliminated since they were based on protocols of upcoming trials. Finally, only 16 studies ^[8–23]^ were selected for this analytical research (Fig. 1).

### 3.2 Description of studies

Sixteen studies with a total number of 25,805 patients (12,891 patients were treated with ticagrelor and 12,914 patients were treated with clopidogrel) were included in this analysis.

A total number of 25,632 patients had ACS (12,794 patients were assigned to the ticagrelor group and 12,838 were assigned to the clopidogrel group) including 12,992 patients who had T segment elevation myocardial infarction (STEMI) (5233 patients were classified in the ticagrelor group versus 7759 patients which were classified in the clopidogrel group) and 14,215 patients who had non-ST segment elevation myocardial infarction (NSTEMI) (6206 patients were treated by ticagrelor versus 8009 patients which were treated by clopidogrel). The remaining participants were patients suffering from stable CAD.

A total number of 23,714 patients with ACS (11,807 patients assigned to the ticagrelor group versus 11,907 patients assigned to the clopidogrel group) were revascularized by PCI.

Patients were enrolled between the years 2004 and 2016. Patients from several corners around the globe especially from regions such as the United States, United Kingdom, Japan, Korea, Spain, Italy, France, Taiwan, and China were included. Other regions were not clearly specified. This current analysis consisted of studies which were published between the years 2007 to 2017. The main features of these studies have been summarized in Table 4.

**Table 4:**
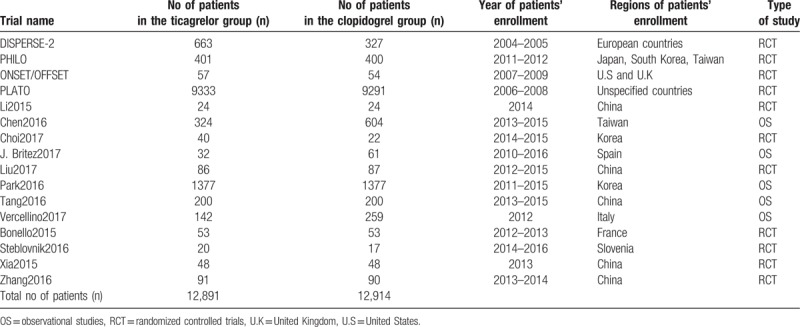
General features of the studies which were included in this analysis.

### 3.3 Baseline characteristics

Baseline features of the patients have been summarized in Table 5. The patients had a mean age which varied between 53.7 and 71.7 years. The percentage of patients with other co-morbidities has been summarized in Table 5.

**Table 5:**
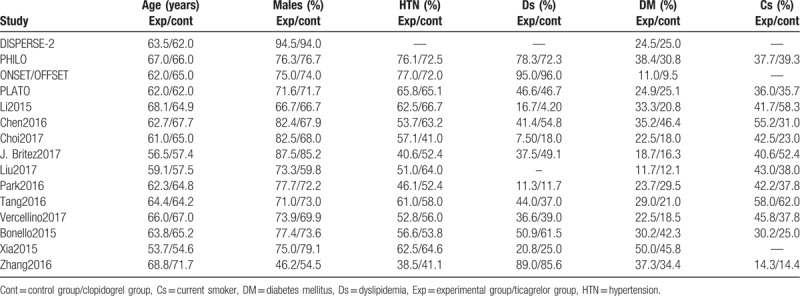
Baseline features of the studies which were included in this analysis.

Other medications such as beta-blockers, angiotensin-converting enzyme inhibitors (ACEI), statins, calcium channel blockers and PPIs have been reported (Table 6).

**Table 6:**
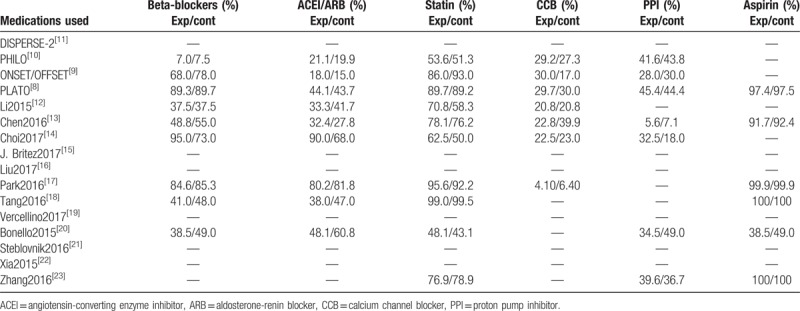
Medication history of the patients.

Overall, no significant difference in baseline features was observed between the 2 groups.

### 3.4 Primary outcomes (outcomes representing efficacy)

**Figure 2:**
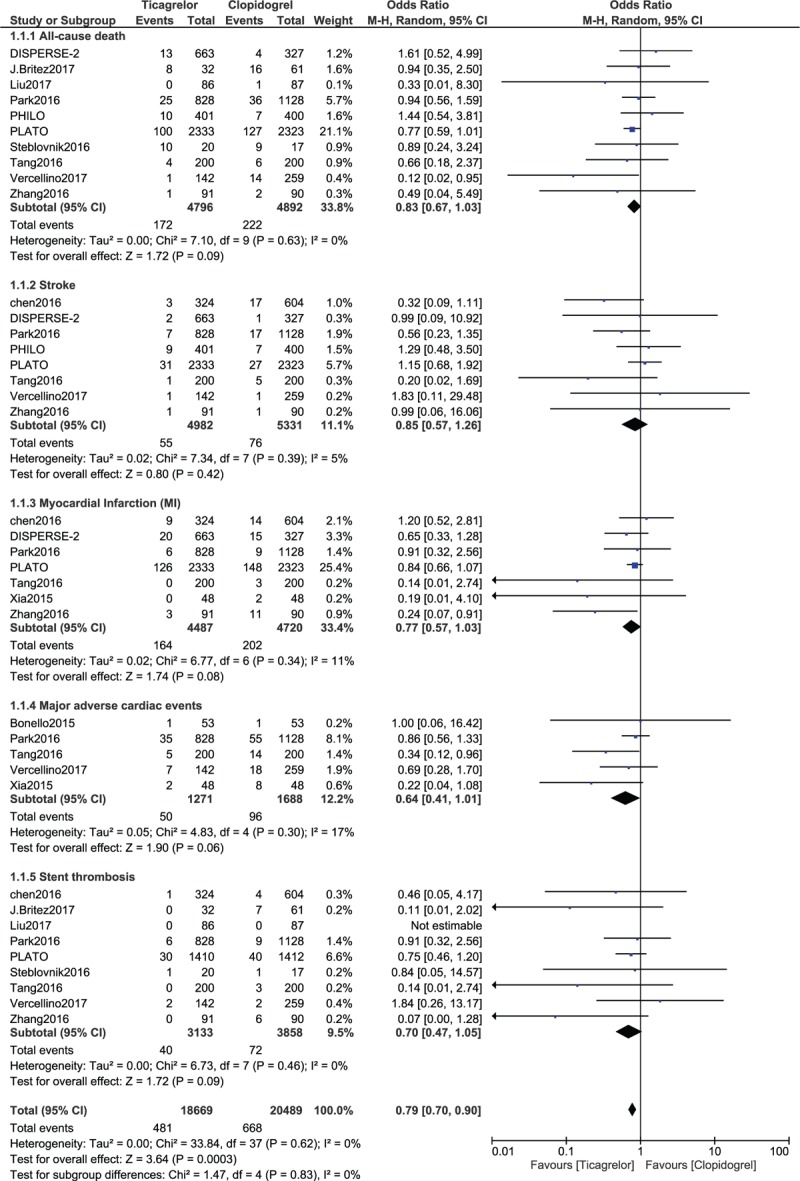
Comparing the efficacy (primary outcomes) observed between Ticagrelor and Clopidogrel.

All-cause mortality, MACEs, MI, stroke, and stent thrombosis were not significantly different with clopidogrel versus ticagrelor with (OR: 0.83, 95% CI: 0.67–1.03; *P* = .09), (OR: 0.64, 95% CI: 0.41–1.01; *P* = .06), (OR: 0.77, 95% CI: 0.57–1.03; *P* = .08), (OR: 0.85, 95% CI: 0.57–1.26; *P* = .42) and (OR: 0.70, 95% CI: 0.47–1.05; *P* = .09) respectively as shown in Figure 2.

### 3.5 Secondary outcomes (outcomes representing safety)

**Figure 3:**
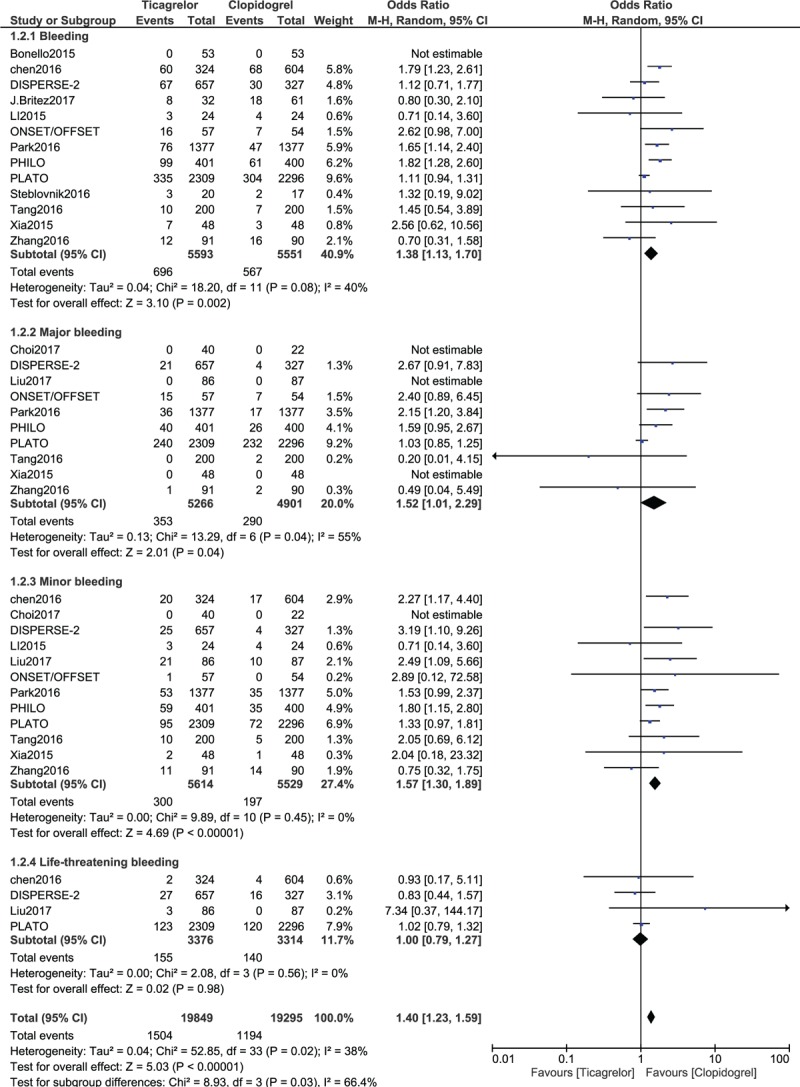
Comparing the bleeding events (secondary outcomes) observed between Ticagrelor and Clopidogrel.

This analysis showed that ticagrelor was associated with a significantly higher rate of overall bleeding (OR: 1.38, 95% CI: 1.13–1.70; *P* = .002) when compared to clopidogrel. When bleeding was further subdivided, minor bleeding was significantly higher with ticagrelor (OR: 1.57, 95% CI: 1.30–1.89; *P* = .00001) as shown in Figure 3. Major bleeding was also significantly higher with ticagrelor (OR: 1.52, 95% CI: 1.01–2.29; *P* = .04). However, life-threatening bleeding (OR: 1.00, 95% CI: 0.79–1.27; *P* = .98) was not significantly different between these 2 antiplatelet drugs (Fig. 3).

**Figure 4:**
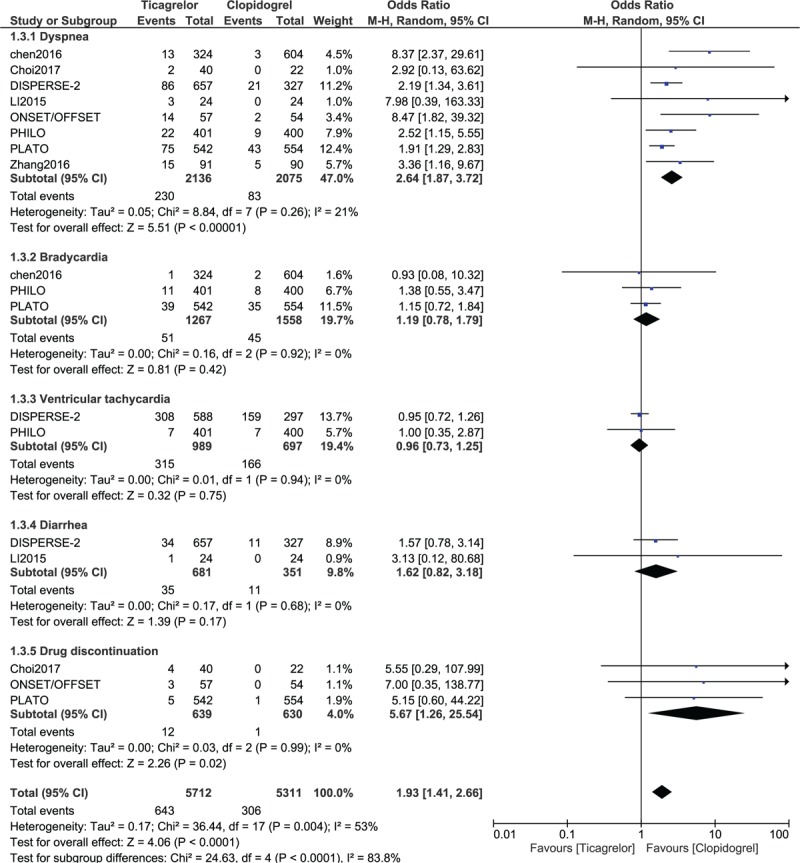
Comparing the adverse drug events (secondary outcomes) observed between Ticagrelor and Clopidogrel.

**Figure 5:**
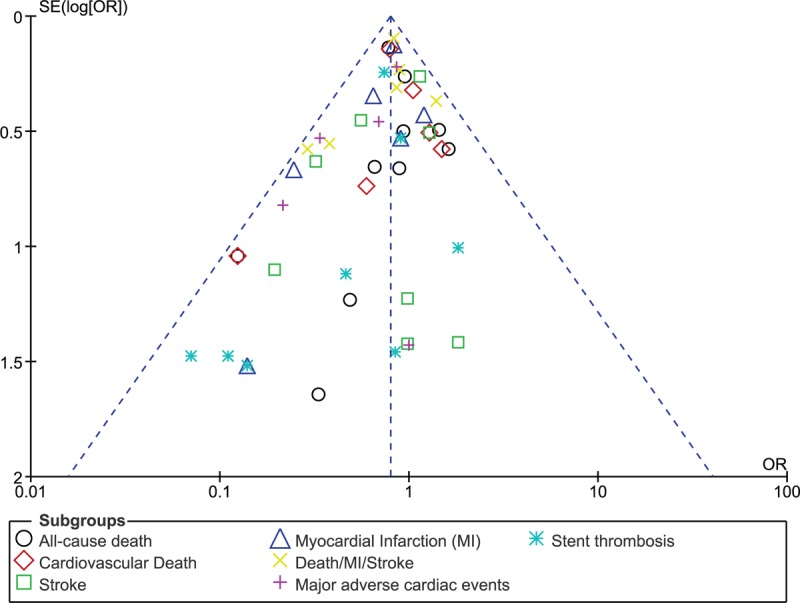
Funnel plot showing publication bias (A).

When the adverse drug events were compared, a significantly higher rate of dyspnea (OR: 2.64, 95% CI: 1.87–3.72; *P* = .00001) was observed in patients who were treated with ticagrelor. However, the results analyzing bradycardia (OR: 1.19, 95% CI: 0.78–1.79; *P* = .42), ventricular tachycardia (OR: 0.96, 95% CI: 0.73–1.25; *P* = .75) and diarrhea (OR: 1.62, 95% CI: 0.82–3.18; *P* = .17) were not significantly different (Fig. 5). Drug discontinuation (OR: 5.67, 95% CI: 1.26–25.54; *P* = .02) was also significantly higher with ticagrelor (Fig. 4).

### 3.6 Sensitivity analyses and publication bias

Sensitivity analyses showed consistent results throughout when each of the studies was excluded 1 by 1 and a new analysis was carried out each time. When sensitivity analysis was carried out on the basis of the duration of follow-up time periods, still consistent results were obtained throughout.

Studies involving patients with stable CAD were excluded and a separate analysis was carried out. Based on the exclusion of patients with stable CAD, results for all-cause mortality (OR: 0.83, 95% CI: 0.67–1.03; *P* = .09), MACEs (OR: 0.64, 95% CI: 0.41–1.01; *P* = .06), stroke (OR: 0.85, 95% CI: 0.57–1.26; *P* = .42), MI (OR: 0.77, 95% CI: 0.57–1.03; *P* = .08) and stent thrombosis (OR: 0.70, 95% CI: 0.47–1.05; *P* = .09) were not significantly different as compared to the results of the main analysis. Bleeding outcomes were also similar to the results of the main analysis.

Another sensitivity subgroup analysis was carried out by excluding all studies which involved participants who did not undergo PCI. Similar results were obtained when compared to the main analysis. All-cause mortality (OR: 0.83, 95% CI: 0.67–1.03; *P* = .09), stroke (OR: 0.96, 95% CI: 0.65–1.42; *P* = .84), MI (OR: 0.72, 95% CI: 0.51–1.00; *P* = .05), MACEs (OR: 0.64, 95% CI: 0.41–1.01; *P* = .06) and stent thrombosis (OR: 0.69, 95% CI: 0.41–1.16; *P* = .16) were not significantly different with ticagrelor or clopidogrel. However, the only difference was that the result representing major bleeding was not significant (OR: 1.43, 95% CI: 0.93–2.20; *P* = .10).

**Figure 6:**
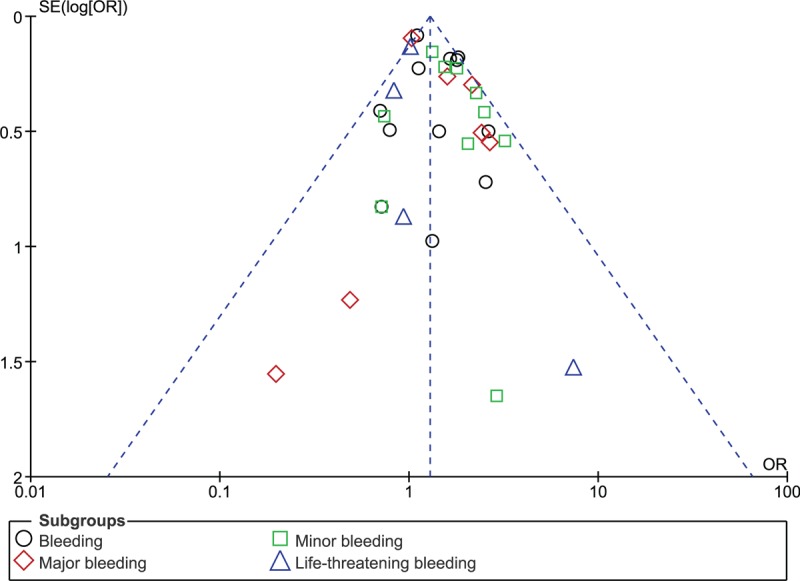
Funnel plot showing publication bias (B).

Based on a visual inspection of the funnel plots which were obtained during the subgroup analyses, there has been a very low evidence of publication bias among the included studies that assessed all the clinical endpoints related to the efficacy and safety observed between ticagrelor and clopidogrel (Figs. 5 and 6).

## 4 Discussion

According to the current results, ticagrelor and clopidogrel were similarly effective in these ACS patients. However, ticagrelor was associated with a significantly higher rate of minor and major bleeding when compared to clopidogrel. Dyspnea was also significantly higher with ticagrelor.

The question which could most probably be asked at this stage would be about the different mechanisms associated with ticagrelor and clopidogrel.

Well, the mechanism of clopidogrel is different from ticagrelor in the way that clopidogrel works irreversibly by inhibiting the receptor P2Y12, an adenosine diphosphate (ADP) on the membrane of platelets, thereby preventing activation of platelets to prevent blood clots^[24]^. Similar to clopidogrel, ticagrelor also inhibits ADP receptors of subtype P2Y12. However, ticagrelor acts differently in the way that it has a binding site which is different from ADP, making its inhibition reversible ^[25]^. Moreover, it does not require hepatic activation of CYP2C19 as compared to clopidogrel. Thus, ticagrelor might result in a faster mode of action compared to clopidogrel.

A sub-study of the PLATO trial comparing the use of ticagrelor versus clopidogrel in patients ≥75 years and in patients <75 years old showed ticagrelor to be associated with significantly lower adverse clinical outcomes compared to clopidogrel.^[26]^ However, the risk of major bleeding was not increased with the use of ticagrelor (hazard ratio 1.02, 95% CI: 0.82–1.27) in these patients with age ≥75 years old and (hazard ratio 1.04, 95% CI: 0.94–1.15). The authors concluded that, in addition to its effectiveness, ticagrelor could also be beneficial in patients with similar age groups. Even in ACS patients with impaired renal function, ticagrelor significantly reduced mortality and other adverse outcomes compared to clopidogrel.^[27]^ However, ticagrelor was associated with non-procedure related bleeding in these patients with chronic kidney disease.

Ticagrelor has also proven to be effective and safe in patients with T2DM.^[28]^ Due to the fact that patients with T2DM are often candidates showing clopidogrel hypo-responsiveness, or have high platelet reactivity, ticagrelor could be another option in this particular subgroup. However, since this current analysis showed a higher minor bleeding and an impending major bleeding associated with ticagrelor, its safety outcomes should further be revised because variation is possible in different subgroups of patients.

The PLATO trial showed significantly lower stent thrombosis associated with ticagrelor in patients with ACS.^[29]^ In addition, in the study published by Cannon et al, which compared ticagrelor with clopidogrel in patients with a planned invasive strategy for ACS, the authors concluded that ticagrelor would be a better option compared to clopidogrel in similar patients for whom an early invasive strategy is planned.^[30]^ However, in this current analysis, no significant difference in stent thrombosis was noted between the 2 antiplatelet agents.

Storey et al, demonstrated the inhibitory effect of ticagrelor and clopidogrel, showing a significantly higher platelet inhibition achieved by the use of ticagrelor whether during the first few hours or during the maintenance treatment period compared to clopidogrel.^[31]^

A recently published meta-analysis showed that major and minor bleeding events were not significantly different with either ticagrelor or clopidogrel.^[32]^ Nevertheless, the study involved only 6 trials and included only studies which were published between the years 2010 and 2015.

To support the results of this analysis, Kang et al, who compared ticagrelor with clopidogrel in Asian and non-Asian patients with ACS did not observe any significant difference in efficacy between ticagrelor and clopidogrel.^[33]^ In addition, a recent meta-analysis comparing newer oral P2Y12 inhibitors versus clopidogrel in patients who were treated for NSTEMI also showed that even if these newer oral P2Y12 inhibitors were more effective by reducing the rate of major adverse events and MI, they were unsafe because they resulted in increased bleeding risks compared to clopidogrel.^[34]^ However, their study assessed ticagrelor and prasugrel together, which would definitely not reflect only the outcomes which were associated strictly with the use of ticagrelor.

Our results have shown ticagrelor to be associated with significantly higher major and minor bleeding in comparison to clopidogrel. A study assessing pharmacodynamics, pharmacokinetics and safety of ticagrelor (50 mg, 100 mg or 200 mg twice daily or 400 mg once daily) showed that a dosage of 100 mg or 200 mg was better than 50 mg twice daily ticagrelor or 75 mg daily clopidogrel and was well tolerated in terms of efficacy.^[35]^ Inhibition of ADP-induced platelet aggregation was measured (final and maximal extent of platelet aggregation) and was shown to be greatest in the 200 mg twice daily or 400 mg once daily ticagrelor group. However, bleeding time was increased to a higher extent in all the ticagrelor groups compared to the clopidogrel group. The study reported 1 major bleeding event (gastro-intestinal bleed resulting in a drop in hemoglobin level) associated with 400 mg ticagrelor. The other dosages of ticagrelor were associated with only minor bleeding events.

However, in patients with ACS, administration of a loading dose has no additive effect on platelet aggregation when switching from ongoing clopidogrel treatment to ticagrelor.^[36]^ Fifty ACS participants who were on dual anti-platelet therapy with aspirin and clopidogrel were randomly assigned to a loading dose of ticagrelor 90 mg and 180 mg respectively (2 separate groups) but no significant difference in platelet aggregation was observed. This might have important clinical implication in the treatment and management of patients with ACS since avoiding a loading dose of ticagrelor might prevent bleeding events. Reasons behind this might be some specific circulating microRNAs ^[37]^sourcing mainly from platelets. Studies have shown a direct correlation with specific level of microRNAs and platelet activation. New research has shown switching from clopidogrel to ticagrelor was associated with significant modulation in the level of specific microRNAs and this might explain the extent of platelet activation in future studies.^[37]^

Nevertheless, whether ticagrelor is really safe compared to clopidogrel will further rely on the POPular AGE study and the TIME trial which will be the first trials to assess bleeding risks between clopidogrel and ticagrelor or prasugrel in elder patients with ACS and to compare the protective effect of clopidogrel and ticagrelor on coronary microvascular dysfunction in patients with ACS respectively.^[38–39]^ In addition, whether the SYNTAX score plays an integral part by contributing to the resulting adverse events should also be further studied.^[40–42]^

## 5 Limitations

First of all, due to the limited number of patients which were assigned to the ticagrelor and clopidogrel groups respectively, the results of this analysis might have been affected. In addition, target vessel revascularization and target lesion revascularization were not reported due to limited data reporting these clinical outcomes. Another limitation of this analysis could be the varied follow-up period following coronary stenting. A few studies which reported only in-hospital outcomes were merged together with the other studies reporting longer follow-up time periods. This could have had an impact on the results which were obtained. The use of other medications including beta-blockers, ACEI/angiotensin-renin blockers, and statin could have had an impact on the outcomes. Moreover, a few studies did not report the number of patients who were on aspirin. Not having included such an important information might contribute to the limitation of this research. In addition, the total number of patients which were extracted from the PLATO trial was reduced to compensate for the small number of patients which were reported in the other studies. Fortunately, sensitivity analyses showed consistent results throughout.

## 6 Conclusion

Ticagrelor and clopidogrel were comparable in terms of efficacy in these patients with ACS. However, the safety outcomes of ticagrelor should further be investigated. Upcoming trials with longer follow-up time periods might be expected to completely solve this issue.

## 7 Authors’ information

Dr WG and Dr HL are co-first authors. From the Department of Cardiology, Jingzhou Central Hospital, the Second Clinical Medical College, Yangtze University, Jingzhou, Hubei, 434020, China.

## Author contributions

WG, HL and KY were responsible for the conception and design, acquisition of data, analysis and interpretation of data, drafting the initial manuscript and revising it critically for important intellectual content. WG and HL wrote the final manuscript.

**Conceptualization:** Wenjun Guan, Hongtao Lu, Keping Yang.

**Data curation:** Wenjun Guan, Hongtao Lu, Keping Yang.

**Formal analysis:** Wenjun Guan, Hongtao Lu, Keping Yang.

**Funding acquisition:** Wenjun Guan, Hongtao Lu, Keping Yang.

**Investigation:** Wenjun Guan, Hongtao Lu, Keping Yang.

**Methodology:** Wenjun Guan, Hongtao Lu, Keping Yang.

**Project administration:** Wenjun Guan, Hongtao Lu, Keping Yang.

**Resources:** Wenjun Guan, Hongtao Lu, Keping Yang.

**Software:** Wenjun Guan, Hongtao Lu, Keping Yang.

**Supervision:** Wenjun Guan, Hongtao Lu, Keping Yang.

**Validation:** Wenjun Guan, Hongtao Lu, Keping Yang.

**Visualization:** Wenjun Guan, Hongtao Lu, Keping Yang.

**Writing – original draft:** Wenjun Guan, Hongtao Lu.

**Writing – review & editing:** Wenjun Guan, Hongtao Lu.
